# *Piper betle* extracts exhibit antitumor activity by augmenting antioxidant potential

**DOI:** 10.3892/ol.2014.2738

**Published:** 2014-11-25

**Authors:** BADRUL ALAM, RAJIB MAJUMDER, SHAHINA AKTER, SANG-HAN LEE

**Affiliations:** 1 Department of Food Science and Biotechnology, Kyungpook National University, Daegu 702-701, Republic of Korea; 2 Department of Pharmacy, Atish Dipankar University of Science and Technology, Dhaka 1213, Bangladesh; 3 Food and Bio-Industry Research Institute, Kyungpook National University, Daegu 702-701, Republic of Korea

**Keywords:** antitumor, antioxidant, Ehrlich ascites carcinoma, *Piper betle*

## Abstract

The present study was conducted to evaluate the methanolic extract of *Piper betle* leaves (MPBL) and its organic fractions with regard to antitumor activity against Ehrlich ascites carcinoma (EAC) in Swiss albino mice and to confirm their antioxidant activities. At 24 h post-intraperitoneal inoculation of tumor cells into mice, extracts were administered at 25, 50 and 100 mg/kg body weight for nine consecutive days. The antitumor effects of the extracts were then assessed according to tumor volume, packed cell count, viable and non-viable tumor cell count, median survival time and increase in life span of EAC-bearing mice. Next, hematological profiles and serum biochemical parameters were calculated, and antioxidant properties were assessed by estimating lipid peroxidation, reduced glutathione (GSH), superoxide dismutase (SOD) and catalase (CAT) levels. MPBL and the ethylacetate fraction (EPBL) at a dose of 100 mg/kg induced a significant decrease in tumor volume, packed cell volume and viable cell count and increased the life span of the EAC-bearing mice (P<0.05). Hematological and serum biochemical profiles were restored to normal levels in the extract-treated mice compared with the EAC control mice. MPBL and EPBL treatment significantly decreased lipid peroxidation (P<0.05) and restored GSH, SOD and CAT levels towards normal compared with the EAC control. Taken together, the results of the present study demonstrated that *Piper betle* extracts exhibit significant antitumor activity, which may be attributed to the augmentation of endogenous antioxidant potential.

## Introduction

Free radicals [reactive oxygen species (ROS) and reactive nitrogen species] are products of normal cellular metabolism, and are extremely reactive and potentially damaging transient chemical species. Numerous endogenous metabolic processes involving redox enzymes and bioenergetic electron transfer generate free radicals, which aid in the conversion of normal cells to cancerous cells (1). ROS are able to affect a number of significant biological molecules, including DNA, proteins and lipids, leading to a number of degenerative diseases, including cancer, Alzheimer’s disease, arthritis and ischemic reperfusion (2). The oxidative damage to DNA may be reduced by antioxidant-rich diets, thus preventing the onset of carcinogenesis (3). In addition, the increasing incidence of cancer and the lack of anticancer drugs has resulted in the pharmacological and chemical investigation of anticancer agents obtained from medicinal plants. At present, >100 novel products obtained from natural sources are in clinical development, particularly as anticancer agents and anti-infectives (4).

Betelvine (*Piper betle*) belongs to the Piperaceae family, which is regarded as a medicinal plant in Southeast Asia. The leaves of *Piper betle* have been found to exhibit wound healing (5), hepatoprotective (6), antioxidant and antifertility effects, as well as antimotility effects on washed human spermatozoa (7). The primary constituent of the leaves is a volatile oil that contains phenols, betel-phenol, chavibetol, chavicol, cadinene and hydroxychavicol, which have been found to exhibit antioxidant and anticarcinogenic activities (8–10). In Bangladesh, the tribal population and aborigines chew these leaves as a narcotic, which causes fainting, profuse sweating and provides body warmth during winter (7).

The present study was performed to evaluate the antioxidant and antitumor activity of the methanolic extract of *Piper betle* leaves (MPBL) and its organic soluble fractions against Ehrlich ascites carcinoma (EAC) in mice.

## Material and methods

### Plant materials

The leaves of *Piper betle* L. were collected from Jahangirnagar University campus, (Dhaka, Bangladesh) in February 2012. The plant material was taxonomically identified by the National Herbarium of Bangladesh (Dhaka, Bangladesh) and recorded as voucher specimen no. JU/3334 for future reference.

### Chemicals

Bovine serum albumin and bleomycin were obtained from Sigma-Aldrich (St. Louis, MO, USA). Trichloroacetic acid was acquired from Merck (Mumbai, India), and thiobarbituric acid (TBA) and nitroblue tetrazolium chloride were purchased from Loba Chemie Pvt. Ltd., (Mumbai, India). 5,5′-Dithiobis(−2-nitro benzoic acid), phenazonium methosulfate, nicotinamide adenine dinucleotide and reduced glutathione (GSH) were purchased from Sisco Research Laboratories Pvt., Ltd., (Mumbai, India). All other chemicals and reagents used were of the highest analytical grade.

### Preparation of plant extract

The plant material was shade-dried with occasional shifting and then ground to a powder using a mechanical grinder, passed through a #40 sieve (mesh size, 0.425 μm) and stored in an air-tight container. A total of 1.0 kg of dried powder material was refluxed with MeOH for 3 h, then the total filtrate was concentrated until dry *in vacuo* at 40°C to render the MeOH extract (240.0 g). The extract was subsequently suspended in dH_2_O and successively partitioned with chloroform (CHCl_3_) and ethylacetate (EtOAc) to supply the CHCl_3_ (90.0 g) and EtOAc (50.0 g) fractions, respectively, and the H_2_O residue (100.0 g).

### Animals

A total of 121 female, 6–7 week-old, Swiss albino mice (weight range, 25–30 g) were used to assess biological activity. The animals were maintained under standard laboratory conditions and had access to food and water *ad libitum*. The animals were acclimatized to the environment for seven days prior to the experimental procedures. All animal experiments were performed in accordance with the guidelines of the Institutional Animal Ethics Committee of Atish Dipankar University of Science and Technology, Dhaka, Bangladesh. Animal treatment and maintenance for acute toxicity and anticancer effects were conducted in accordance with the Principle of Laboratory Animal Care (NIH publication No. 85-23, revised 1985) and the Animal Care and Use Guidelines of Atish Dipankar University of Science and Technology.

### Acute toxicity study

An acute oral toxicity assay was performed using healthy, non-pregnant, adult female, Swiss albino mice (weight range, 25–30 g) divided into six different groups. Increasing oral doses of MPBL (50, 100, 200, 500 and 1,000 mg/kg body weight) in distilled water were administered at 20 ml/kg to the different test groups. The normal group received distilled water only. Following treatment, the mice were allowed to feed *ad libitum* and observed for 48 h for any mortality or behavioral changes (11).

### Tumor transplantation

EAC cells were obtained from the Indian Institute of Chemical Biology (Calcutta, India). The EAC cells were maintained *in vivo* in Swiss albino mice by intraperitoneal (i.p.) transplantation of 2×10^6^ cells per mouse every 10 days. Ascitic fluid was drawn from the EAC tumor-bearing mice at the log phase (days 7–8 of tumor-bearing) of the tumor cells and each test animal received 0.1 ml of i.p. tumor cell suspension containing 2×10^6^ tumor cells.

### Treatment schedule

The animals were divided into eight groups (n=12) and provided with food and water *ad libitum*. All animals in each group received EAC cells (2×10^6^ cells/mouse i.p.) with the exception of group-I, which served as the normal saline control (5 ml/kg body weight i.p.). Group II served as the EAC control. At 24 h post-EAC transplantation, groups III, IV and V received MPBL at doses of 25, 50 and 100 mg/kg i.p., respectively. Groups VI and VII also received CHCl_3_ (CPBL) and EtOAc (EPBL) extract at doses of 100 mg/kg i.p., whereas group VIII, serving as a positive control, received bleomycin (0.3 mg/kg i.p) for nine consecutive days (12). At 24 h post-administration of the last dose, the animals were fasted for 18 h, at which point, six animals in each group were sacrificed by cardiac puncture for the estimation of hematological and serum biochemical parameters, and to measure antitumor and liver biochemical parameters. The remainder were provided with food and water *ad libitum* and observed to determine if there were any changes in lifespan. The antitumor activity of the extract was measured in the EAC animals as described next.

### Determination of tumor and packed cell volume

The mice were dissected and ascitic fluid was collected from the peritoneal cavity. The tumor volume was measured using a graduated centrifuge tube and the packed cell volume was determined by centrifuging the fluid at 1,000 × g for 5 min.

### Viable and non-viable tumor cell count

The ascitic fluid was collected in a white blood cell (WBC) pipette and diluted 100 times. A drop of the diluted suspension was then placed on a Neubauer counting chamber (Celeromics, Cambridge, UK) and the cells were stained with Trypan blue (0.4% in normal saline). The cells that did not take up the dye were considered viable, while those that did were considered non-viable. The viable and non-viable cells were then counted using the following formula: Cell count = (number of cells × dilution factor)/(area × thickness of liquid film).

### Determination of median survival time (MST) and percentage increase in life span

The rate of mortality was monitored by recording the percentage increase in life span (% ILS) and MST according to the following formula (13): MST in days = (day of first mortality + day of last mortality)/2.

### Estimation of hematological and serum biochemical parameters

Blood was collected to estimate the hemoglobin (Hb) content, and red blood cell (RBC) and WBC counts (12). Differential counts of WBCs were performed from Leishmen-stained blood smears (13). Serum biochemical parameters, including serum glutamate oxaloacetate transaminase (SGOT), serum glutamate pyruvate transaminase (SGPT) (14), serum alkaline phosphatase (SALP), serum bilirubin (15) and total protein (16) levels were also estimated.

### Estimation of lipid peroxidation thiobarbituric acid reactive substances (TBARS)

The TBARS in the liver tissue were measured as described by Ohkawa *et al* (17) and expressed as μmols of malondialdehyde (MDA)/g of liver tissues.

### Estimation of reduced GSH level

The GSH level of liver tissue was determined as described by Ellman (18) and expressed as μg/g of liver tissues.

### Estimation of superoxide dismutase (SOD) and catalase (CAT) levels

The SOD and CAT activity in the liver tissue was analyzed according to the methods described by Pari and Latha (19). The SOD activity was expressed as U/mg of liver tissue and CAT was expressed in terms of μmol of hydrogen peroxide decomposed/min/mg of liver tissue.

### Statistical analysis

All values are expressed as the mean ± standard error of the mean of three replicate experiments. Statistical analysis was performed using the SPSS version 16.0 software (SPSS Inc., Chicago, IL, USA). All *in vivo* data were assessed using analysis of variance followed by Dunnett’s test and P<0.05 was considered to indicate a statistically significant difference.

## Results

### Acute toxicity studies

Acute toxicity studies are primarily designed to develop therapeutic indices, for example, the ratio between the pharmacologically effective dose and lethal dose against the same strain and species. MPBL was safe at doses as high as 1,000 mg/kg [*per os* (p.o.)] body weight, causing no mortality, behavioral changes, locomotor ataxia, diarrhea or weight loss in mice during 48 h of observation. Additionally, food and water intake did not differ among the groups studied (data not shown).

### Tumor growth and survival parameters

**Figure 1:**
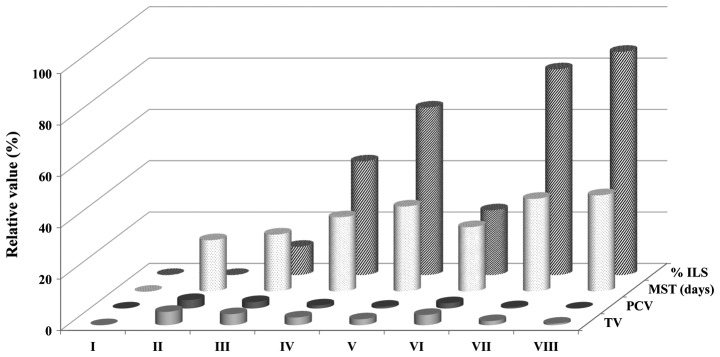
Effects of *Piper betle* extract on tumor volume, packed cell volume, MST and % ILS in EAC-bearing mice. The data are presented as the mean ± standard error of the mean (n=12 mice per group). ^*^P<0.05 vs. EAC control group. Group I animals received normal saline (5 ml/kg), whereas group II animals received EAC control (2×10^6^ cell/mouse), group VIII received bleomycin, 0.3 mg/kg body weight, and groups III, IV and V, were treated with 25. 50 and 100 mg/kg body weight (p.o.) of the MPBL, respectively. Groups VI and VII were treated with 100 mg/kg body weight (p.o.) of the CPBL and EPBL, respectively. EAC, Ehrlich ascites carcinoma; TV, total volume; PCV, packed cell volume; MST, mean survival time; % ILS, percentage increase in life span; p.o., per os; MPBL, methanolic extract of *Piper betle* leaves; CPBL, chloroform; EPBL,. ethylacetate fraction.

MPBL and EPBL at a dose of 100 mg/kg body weight significantly reduced the body weight, tumor volume, packed cell volume and viable tumor cell count (Fig. 1), however, the non-viable tumor cell count was increased compared with the EAC control group (data not shown). However, CPBL exerted no significant effects at a dose of 100 mg/kg. The MST increased to 22.31±0.11 (% ILS, 10.99), 29.01±0.17 (% ILS, 44.25) and 33.23±0.21 days (% ILS, 65.25) following the administration of MPBL at doses of 25, 50 and 100 mg/kg body weight, respectively, while the EPBL and the reference drug, bleomycin, exhibited survival times of 36.23±0.31 (% ILS, 80.15) and 37.60±0.11 days (% ILS, 86.97), respectively (Fig. 1). Finally, the change in body weight (data not shown) of the animals indicated that *Piper betle* extracts had the potential to inhibit tumor growth.

### Hematological parameters

**Figure 2:**
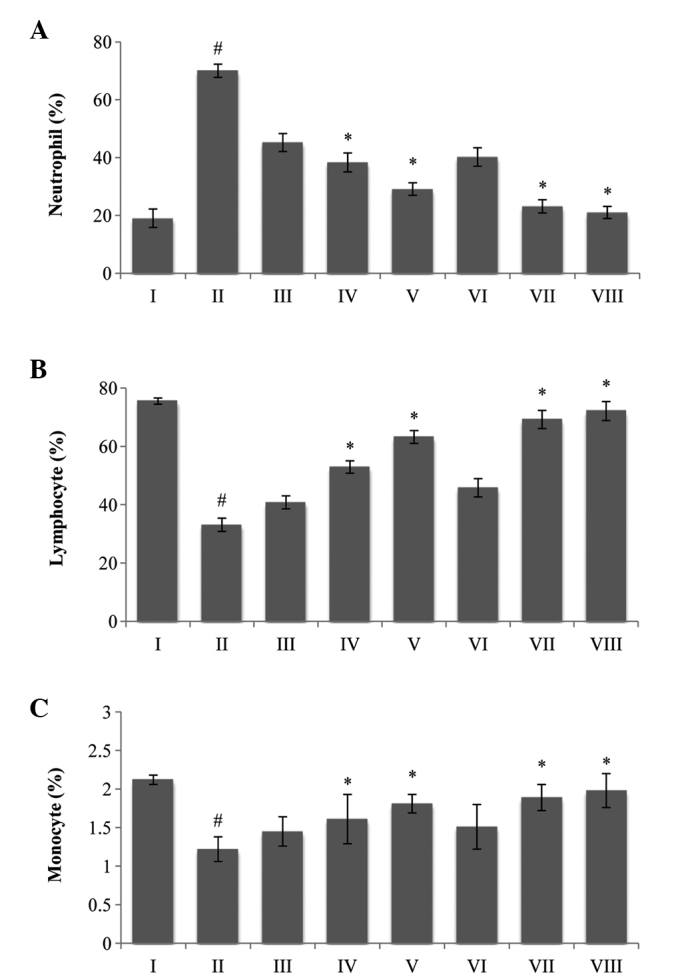
A classical feature of *Piper betle* extracts on differential count of WBCs; (A) neutrophils, (B) lymphocytes and (C) monocytes. The data are presented as the mean ± standard error of the mean. (n=6 mice per group), ^#^P<0.05 vs. normal saline group.^*^P<0.05 vs. EAC control group. Group I animals received normal saline (5 ml/kg), whereas group II received EAC control (2×10^6^ cell/mouse), group VIII received bleomycin, 0.3 mg/kg body weight, and groups III, IV and V were treated with 25, 50 and 100 mg/kg body weight (p.o.) of MPBL, respectively. Groups VI and VII were treated with 100 mg/kg body weight (p.o.) of CPBL and EPBL, respectively. EAC, Ehrlich ascites carcinoma; p.o., *per os*; MPBL, methanolic extract; CPBL, chloroform; EPBL, ethylacetate fraction.

The hematological parameters of the tumor-bearing mice were found to be significantly different compared with the normal group. The total WBC count increased (P<0.05) and the Hb content and RBC count decreased in the EAC control animals compared with the normal saline group. Treatment with MPBL and EPBL at a dose of 100 mg/kg body weight significantly increased the Hb content and RBC count towards normal levels (data not shown). Additionally, the number of neutrophils (Fig. 2A) was increased, while the numbers of lymphocytes (Fig. 2B) and monocytes (Fig. 2C) were found to decrease in the EAC control group compared with the normal group. These results indicated that treatment with varying doses of *Piper betle* extract could significantly change these altered parameters to near normal values (Fig. 2).

### Effect on biochemical parameters

As shown in Table I, the biochemical parameters, including SGOT, SGPT, SALP and bilirubin levels, in the EAC control group were significantly upregulated compared with the normal group. Treatment with MPBL and EPBL at doses of 100 mg/kg significantly decreased the SGOT, SGPT, SALP and bilirubin levels to approximately normal levels, whereas CPBL at this dose did not produce the optimum results. The total protein level was significantly lower in the EAC control group compared with the normal group (P<0.05). The administration of MPBL and EPBL at a dose of 100 mg/kg body weight in the EAC-bearing mice led to a significant increase in total protein level compared with the EAC control group.

### Effect on lipid peroxidation and reduced GSH

**Figure 3:**
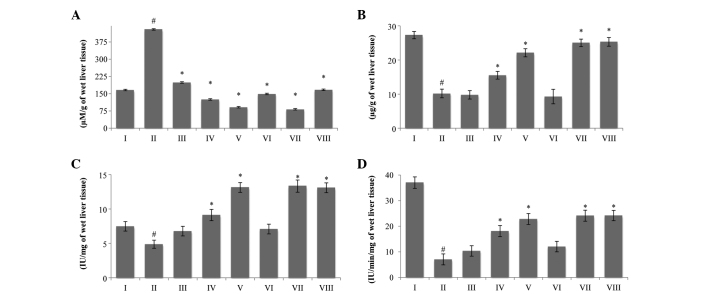
Effect of *Piper betle* on (A) lipid peroxide, (B) reduced glutathione, (C) superoxide dismutase and (D) catalase levels in EAC-bearing mice. The data are presented as the mean ± standard error of the mean (n=6 mice per group), ^#^P<0.05 vs. normal saline group.^*^P<0.05 vs. EAC control group. Group I animals received normal saline (5 ml/kg), whereas group II received EAC control (2×10^6^ cell/mouse), group VIII received bleomycin, 0.3 mg/kg body weight, and groups III, IV and V were treated with 25, 50 and 100 mg/kg body weight (p.o.) of MPBL, respectively. Groups VI and VII were treated with 100 mg/kg body weight (p.o.) of CPBL and EPBL, respectively. EAC, Ehrlich ascites carcinoma; p.o., *per os*; MPBL, methanolic extract; CPBL, chloroform; EPBL, ethylacetate fraction.

Following administration of 100 mg/kg MPBL or EPBL, or 0.3 mg/kg bleomycin to the EAC-bearing mice, the level of lipid peroxidation decreased by 91.34±1.10, 82.34±1.10 and 167.21±1.04 μM/g, respectively, compared with the EAC control group (166.19±3.06 μM/g of wet liver tissue; P<0.05; Fig. 3A). Reduced GSH levels (39 μg/g of wet liver tissue) were found to be significantly elevated towards the normal level upon administration of MPBL and EPBL at 100 mg/kg compared with the EAC control group (P<0.05; Fig. 3B).

### Effect on SOD and CAT

The administration of MPBL at a dose of 25, 50 and 100 mg/kg markedly increased the levels of SOD and CAT in a dose-dependent manner (P<0.05; Fig. 3C and D) compared with the EAC control group. By contrast, EPBL exhibited almost the same activity as standard bleomycin for the two parameters (Fig. 3).

## Discussion

Initially reported as a spontaneous murine mammary adenocarcinoma, the Ehrlich tumor can be grown in the majority of mouse strains and is accepted as a transplantable tumor model to examine the antitumor effects of a number of substances (20).

In the present study, a rapid increase in ascitic tumor volume was observed in EAC tumor-bearing mice and the treatment with *Piper betle* extracts reduced the intraperitoneal tumor burden, thereby reducing the tumor volume, tumor weight and viable tumor cell count, while increasing the life span of the tumor-bearing mice. Therefore, it may be hypothesized that the increase in lifespan of EAC-bearing mice in response to MPBL and EPBL at 100 mg/kg may be due to a decrease in nutritional fluid volume and a delay in cell division (12). Reductions in viable cell count and increased non-viable cell count towards normal in tumor hosts indicate antitumor effects against EAC cells in mice. These results indicate that MPBL and EPBL have a direct association with tumor cells at higher doses as they absorb the anticancer drug by direct absorption in the peritoneal cavity, resulting in lysis of the cells via a direct and cytotoxic mechanism. Anemia and myelosuppression have frequently been observed in ascites carcinoma due to a deficiency in iron, in hemolytic or myelopathic conditions, resulting in a reduced number of RBCs (21). In the present study, treatment with *Piper betle* extracts returned the hemoglobin content and RBC and WBC counts to almost normal levels (data not shown), indicating that the extracts exhibit hematopoietic protecting activity without myelotoxicity, the most common side-effect of cancer chemotherapy.

A preliminary phytochemical study indicated the presence of alkaloids, steroids, tannins, phenolic and flavonoid compounds and glycosides in crude extracts of *Piper betle* (7). A number of studies have indicated that the presence of steroids, terpenoids and phenolic compounds, including coumarins, tannins and flavonoids, exert a chemopreventive role in the progression of cancer by affecting signal transduction in cell proliferation and angiogenesis (22). Incorporation of phytosterols into the cell membrane can alter the fluidity of membranes and the activity of membrane-bound enzymes. In addition, phytosterols cause alterations in pathway signal transduction, resulting in the growth of tumors and the stimulation of apoptosis in tumor cell lines. Phytosterols have been demonstrated to enhance the *in vitro* proliferation of human peripheral blood lymphocytes and T cells, indicating the possible stimulation of the immune system (23). The marked anticancer activities of MPBL and EPBL are possibly due to the presence of alkaloids, phenolic compounds, flavonoids and terpenoids, and their synergistic effects.

ROS exhibit multiple functions and are involved in tumor initiation and progression (24). MDA, a free oxygen radical product formed during oxidative degeneration of cancerous tissues (25) and as the end product of lipid peroxidation, is a biomarker of oxidative stress that has been reported to be exhibited at higher levels in cancer tissues than in non-diseased organs (26). The results of the present study indicated that the TBARS levels in the cancerous tissues were higher than those in the normal tissues (Fig. 3). Treatment with EPBL inhibited hepatic lipid peroxidation, as indicated by the reduction of MDA levels toward normal levels, emphasizing the reduction in free radical production and the subsequent decrease in damage to the cell membrane and MDA production in the tumor-bearing mice.

Depleted endogenous antioxidant enzyme levels with enhanced free radical generation have been well documented in carcinogenesis (27). Numerous tumor cells with pro-oxidant status promote oxidative stress, which increases the surviving potential of the cancer cells by inducing mutations, activating redox signaling and stimulating pro-survival factors, such as nuclear factor-κB and activator protein-1 (28). GSH, which strongly inhibits the neoplastic process, is important in the endogenous antioxidant system. This compound acts mainly as a reducing agent and detoxifies hydrogen peroxide when GSH peroxidase is present (29). In the current study, the GSH levels in the experimental mice were found to be significantly lower than those in the EAC control mice (Fig. 3). These results revealed that the antitumor activity of MPBL and EPBL was accompanied by the enhancement of non-enzymatic antioxidant protection.

It is well-known that cells exhibit enzymatic antioxidant mechanisms, such as the generation of SOD and CAT, which are involved in the elimination of free radicals (30). SOD and CAT are involved in the scavenging of superoxide and hydrogen peroxide. In a previous study, decreased levels of SOD activity were detected in EAC-bearing mice in response to the loss of Mn^2+^-SOD activity and mitochondria in EAC cells, resulting in a decrease in the amount of total SOD activity in the liver (31). The inhibition of SOD and CAT activity as a consequence of tumor growth has also been reported (32). Similar findings were observed in the present study on EAC-bearing mice. The administration of MPBL and EPBL at higher doses increased the SOD and CAT levels towards normal levels.

Plant-derived extracts with antioxidant potential have demonstrated cytotoxicity against tumor cells and antitumor activity in experimental animals (33). The cytotoxic and antitumor activity of plant-derived products occurs either through the induction of apoptosis or the inhibition of neovascularization (34). In the present study, higher doses of MPBL and EPBL dramatically reduced tumor growth and the viability of the tumor cells, and normalized the hematological and serum biochemical profiles, increasing the life span compared with the EAC control mice. MPBL and EPBL treatment improved the endogenous non-enzymatic and enzymatic antioxidant systems (Fig. 3). The decrease in lipid peroxidation and the elevation of GSH, SOD and CAT levels in the MPBL- and EPBL-treated mice indicated the potential of *Piper betle* extract as an inhibitor of EAC-induced intracellular oxidative stress.

In conclusion, *Piper betle* leaf extracts exhibited marked antitumor activity against EAC in the mice of the present study, possibly by modulating lipid peroxidation and augmenting endogenous antioxidant defense systems. Future studies are required to investigate the isolation and characterization of lead compounds responsible for the aforementioned activity of this plant.

## Figures and Tables

**Table I:** Effects of *Piper betle* on serum biochemical parameters in EAC-bearing mice.

Group, n	SGOT, IU/l	SGP, IU/l	SALP, IU/l	Total protein, mg/dl	Bilirubin, mg/dl
I	38.32±1.41	28.02±4.32	77.91±2.24	9.67±0.24	0.91±0.19
II	74.12±1.11^a^	66.32±5.32^a^	120.11±3.24^a^	5.78±0.14^a^	3.75±0.12^a^
III	65.52±3.51^b^	52.12±5.12^b^	108.31±1.24^b^	6.08±0.19^b^	2.85±0.75^b^
VI	42.52±5.11^b^	43.32±2.32^b^	97.19±5.24^b^	6.18±0.34^b^	2.15±0.18^b^
V	38.12±1.01^b^	39.39±1.12^b^	88.10±1.27^b^	6.98±1.34^b^	1.75±1.18^b^
VI	55.52±3.51^b^	48.12±5.12^b^	101.31±1.24^b^	6.78±0.19^b^	2.35±0.75^b^
VII	34.12±1.01^b^	36.39±1.12^b^	83.10±1.27^b^	7.78±1.34^b^	1.25±1.18^b^
VIII	35.13±1.91^b^	33.01±1.31^b^	73.90±1.92^b^	8.38±1.14^b^	0.98±1.18^b^

Data is presented as mean ± standard error of the mean (n=6 mice per group).

a P<0.05 vs. normal saline group.

b P<0.05 vs. EAC control group.

Group I animals were administered normal saline (5 ml/kg), whereas group II received EAC control (2×106 cell/mouse), group VIII received bleomycin, 0.3 mg/kg body weight, and groups III, IV and V were treated with 25, 50 and 100 mg/kg body weight (p.o.) of MPBL, respectively. Groups VI and VII were treated with 100 mg/kg body weight (p.o.) of CPBL and EPBL, respectively. EAC, Ehrlich ascites carcinoma; p.o., per os; SGOT, serum glutamate oxaloacetate transaminase; SGPT, serum glutamate pyruvate transaminase; SALP, serum alkaline phosphatase.
